# CdtR Regulates TcdA and TcdB Production in *Clostridium difficile*

**DOI:** 10.1371/journal.ppat.1005758

**Published:** 2016-07-14

**Authors:** Shelley A. Lyon, Melanie L. Hutton, Julian I. Rood, Jackie K. Cheung, Dena Lyras

**Affiliations:** Infection and Immunity Program, Biomedicine Discovery Institute and Department of Microbiology, Monash University, Clayton, Victoria, Australia; University of Illinois, UNITED STATES

## Abstract

*Clostridium difficile* is a global health burden and the leading cause of antibiotic-associated diarrhoea worldwide, causing severe gastrointestinal disease and death. Three well characterised toxins are encoded by this bacterium in two genetic loci, specifically, TcdB (toxin B) and TcdA (toxin A) in the Pathogenicity Locus (PaLoc) and binary toxin (CDT) in the genomically distinct CDT locus (CdtLoc). Toxin production is controlled by regulators specific to each locus. The orphan response regulator, CdtR, encoded within the CdtLoc, up-regulates CDT production. Until now there has been no suggestion that CdtR influences TcdA and TcdB production since it is not carried by all PaLoc-containing strains and CdtLoc is not linked genetically to PaLoc. Here we show that, in addition to CDT, CdtR regulates TcdA and TcdB production but that this effect is strain dependent. Of clinical relevance, CdtR increased the production of TcdA, TcdB and CDT in two epidemic ribotype 027 human strains, modulating their virulence in a mouse infection model. Strains traditionally from animal lineages, notably ribotype 078 strains, are increasingly being isolated from humans and their genetic and phenotypic analysis is critical for future studies on this important pathogen. Here we show that CdtR-mediated toxin regulation did not occur in other strain backgrounds, including a ribotype 078 animal strain. The finding that toxin gene regulation is strain dependent highlights the regulatory diversity between *C*. *difficile* isolates and the importance of studying virulence regulation in diverse lineages and clinically relevant strains. Our work provides the first evidence that TcdA, TcdB and CDT production is linked by a common regulatory mechanism and that CdtR may act as a global regulator of virulence in epidemic 027 strains.

## Author Summary

*Clostridium difficile* is the leading cause of antibiotic-associated diarrhoea. The TcdB, TcdA and binary toxins produced by *C*. *difficile* are encoded in two genomically distinct loci: TcdB and TcdA in the Pathogenicity Locus (PaLoc) and binary toxin (CDT) in the CDT locus (CdtLoc). Toxin production is primarily controlled by regulators specific to each locus. Because the presence of these loci varies amongst different strains of *C*. *difficile*, no rational link for their co-regulation has ever been proposed. Here we have shown that the regulator of CDT production, CdtR, also regulates production of TcdA and TcdB in a strain dependent manner. These results represent the first evidence that TcdA and TcdB production is linked to the production of CDT by a common regulatory mechanism. Collectively, our results establish CdtR as an important virulence regulator in two clinically important, epidemic strains of *C*. *difficile*, and further highlights the need to investigate regulatory mechanisms of important virulence factors in diverse strain backgrounds.

## Introduction

**Fig 1:**
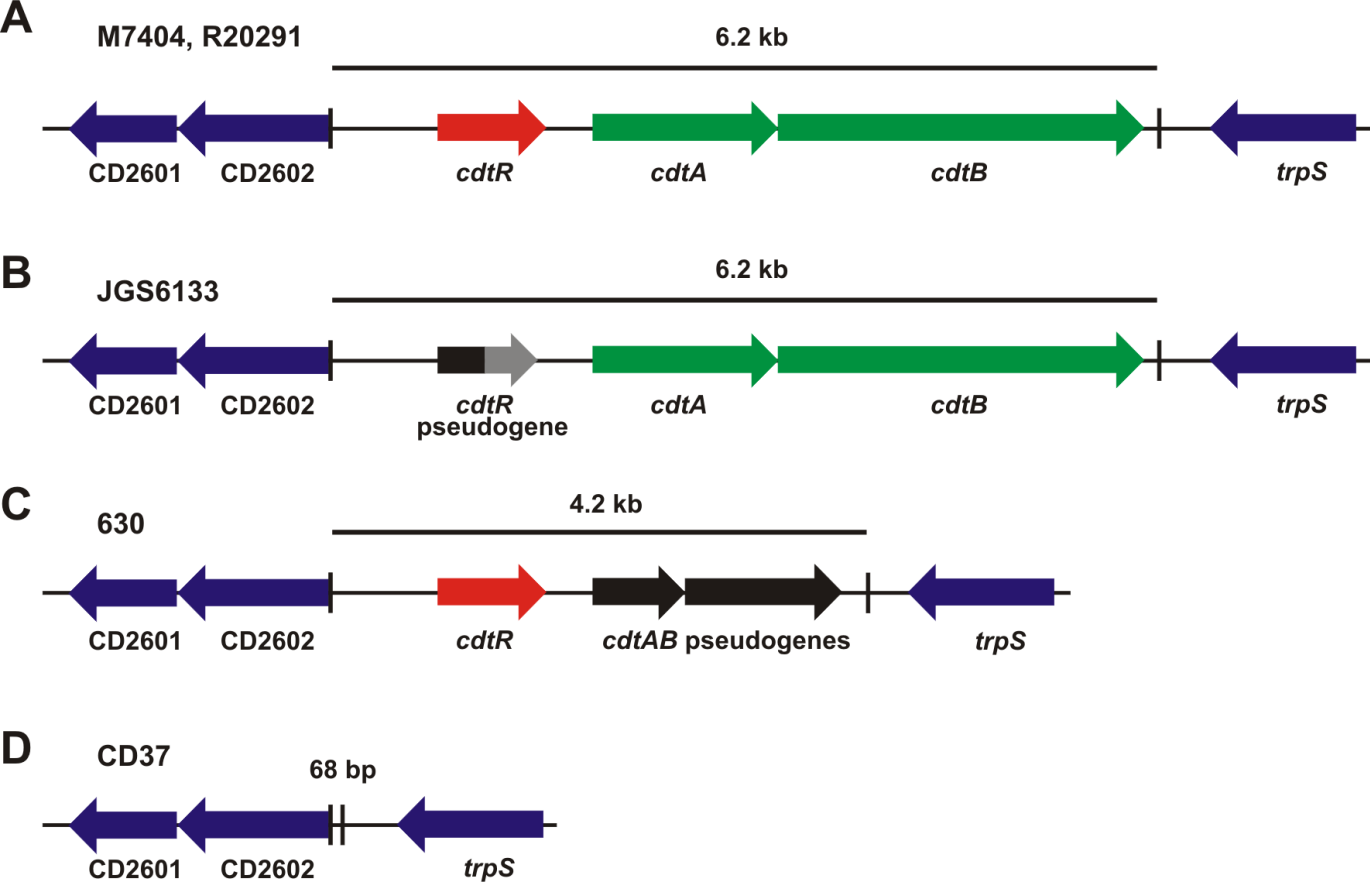
Schematic representation of the CDT loci from representative strains. **a,** The full length CdtLoc from the ribotype 027 strains (M7404 and R20291). **b,** The CdtLoc from the ribotype 078 strain, JGS6133. The *cdtR* pseudogene is shown in black and is grey after the premature stop codon. **c,** The CdtLoc from the ribotype 012 strain 630 carrying *cdtAB* pseudogenes (shown in black). **d,** The CdtLoc in the CDT negative strain CD37 is replaced with a 68 bp sequence. The boundaries of the CdtLoc are indicated with vertical lines and the flanking genes are blue.

*C*. *difficile* antibiotic-associated diarrhoea is a toxin mediated disease [1,2]. During infection, TcdA, TcdB and CDT are secreted into the colonic epithelium by this bacterium, leading to diarrhoea that can progress to serious, life threatening inflammatory diseases, including pseudomembranous colitis and toxic megacolon [3]. The production of these toxins varies between strains. TcdB is the most commonly encoded toxin and is most often co-located with the TcdA gene in the PaLoc region [4], both toxins act as monoglucosyltransferases that irreversibly modify Rho family members [3]. PaLoc variants that produce TcdB and not TcdA are, however, becoming increasingly common, for example, they represented 23% of strains in one recent study of human strains in China [5]. CDT is encoded in a specific locus, CdtLoc (Fig 1) [6] the carriage of which has also increased significantly over the last decade; in 2004 6% of clinical isolates encoded CDT whereas 33.5% now encode this toxin [7,8]. CDT is an ADP-ribosyltransferase that is not essential for disease, but may be important for colonisation during an infection [9]. CdtLoc is not carried by all PaLoc-containing strains and it is not linked genetically to PaLoc.

Regulation of toxin production in *C*. *difficile* is somewhat strain dependent, suggesting that toxin regulatory mechanisms have evolved independently to modulate pathogenesis [10–13]. The TcdR alternative sigma factor and TcdC anti-sigma factor, which are encoded with *tcdA* and *tcdB* in the PaLoc, act as the primary mechanism controlling the production of these toxins [14,15]. TcdA and TcdB regulation has also been linked to many important cellular processes in the *C*. *difficile* life cycle, including sporulation, by Spo0A, the master sporulation regulator, motility, *via* the flagella regulator SigD, and nutrient acquisition, by the regulators of carbon and amino acid metabolism, CcpA and CodY [10,11,16–20]. The ribotype 027 strains associated with epidemics of severe CDI appear to be more virulent than strains previously isolated, a phenotype that has been partly attributed to increased TcdA and TcdB production [21–23]. By comparison, little is known about the regulation of CDT production beyond the involvement of CdtR, and until now, no link had been identified between the control of CDT, TcdA and TcdB production. The difficulty in genetically manipulating strains from different *C*. *difficile* clonal lineages has also prevented a broader analysis of the role of this regulator across different strain types.

In this study, we investigated the role of CdtR in different strains of *C*. *difficile* including two epidemic ribotype 027 strains. As expected, CdtR was found to regulate the production of CDT. Surprisingly, however, CdtR also regulated the production of the PaLoc encoded toxins, TcdA and TcdB, in the two ribotype 027 strains. Further analysis showed that regulation occurred at the transcriptional level and probably resulted from indirect regulation of the positive regulator of PaLoc gene expression, TcdR. Importantly, further analysis showed the importance of CdtR for *C*. *difficile* pathogenesis, with *cdtR* mutants causing less severe disease than the wild type strain in a mouse infection model. To determine whether CdtR function is conserved across evolutionarily diverse isolates, ribotype 078 (JGS6133) and 012 (630) strains were investigated. CdtR regulated CDT production in the ribotype 078 strain; the ribotype 012 strain does not encode CDT. Notably, and in contrast to the ribotype 027 strains, CdtR did not regulated TcdA or TcdB production in either strain background, highlighting the regulatory variation of key virulence factors between *C*. *difficile* strains. These results highlight the importance of investigating regulatory mechanisms in clinically important strains of *C*. *difficile* and suggest that CdtR-mediated toxin regulation is an important virulence mechanism in the epidemic ribotype 027 strains.

## Results

### CdtR regulates production of CDT, TcdA and TcdB in two epidemic, ribotype 027 strains of *C*. *difficile*

**Fig 2:**
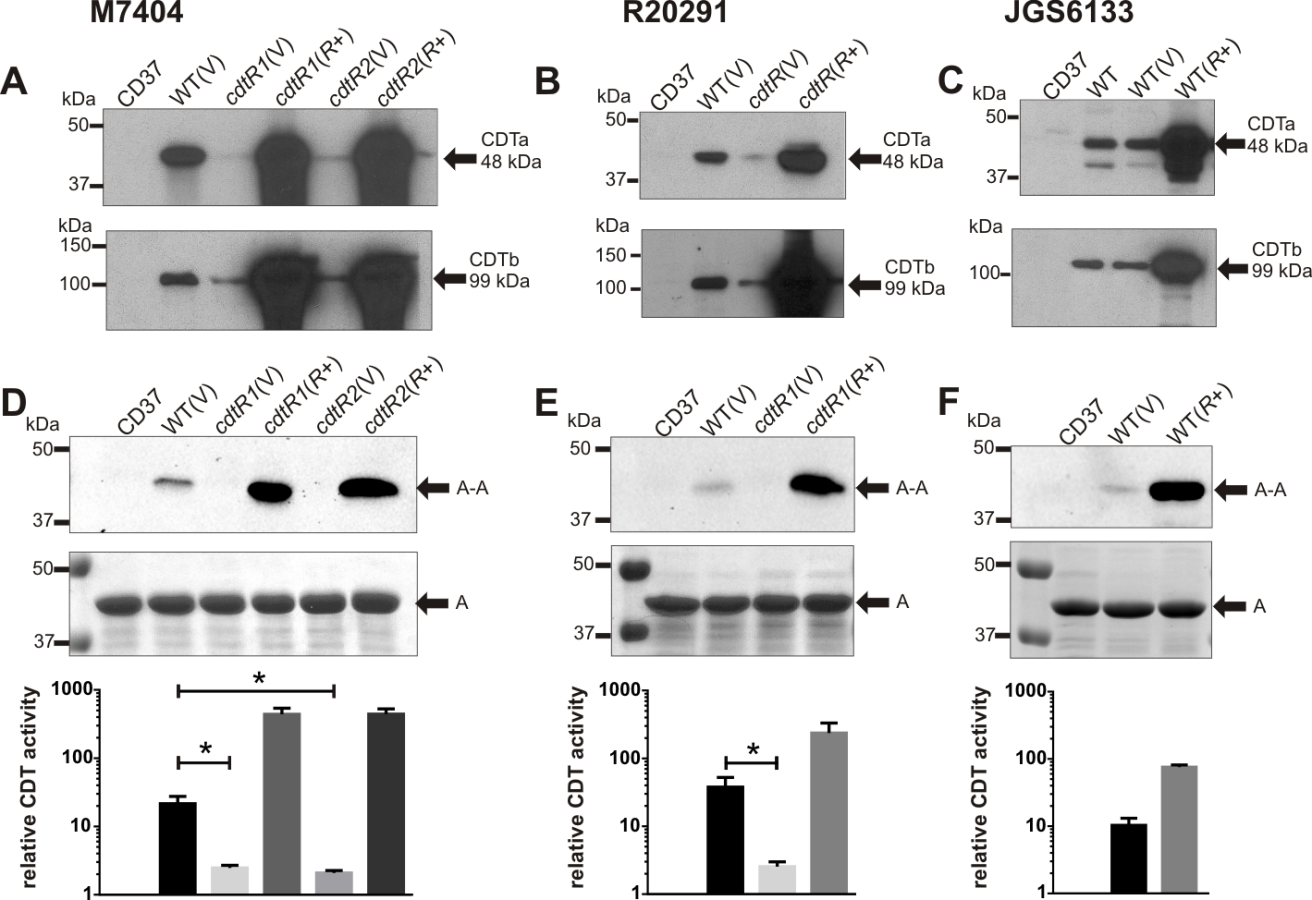
Analysis of CDT production. **a–c,** Western immunoblot using CDTa-specific and cross-reactive Ib-specific antibodies and precipitated supernatants from the strains indicated. CD37 (non-toxigenic), V = vector control, *R*+ = *cdtR* complemented. The arrows indicate the 48 kDa CDTa and 99 kDa CDTb proteins. **d–f,** CDT activity assessed by ADP-ribosyltransferase assay. Samples were separated by SDS-PAGE and biotinylated (ADP-ribosylated) actin detected by HRP-streptavidin. Relative CDT activity was assessed by densitometry compared to the non-toxigenic control strain CD37. A = actin, A-A = ADP-ribosylated actin. Data represent the mean ± SEM (n = 3). *, p ≤ 0.05.

To investigate the role of CdtR in the regulation of CDT production in the epidemic ribotype 027 strains, we constructed two independent *cdtR* mutants in the Canadian isolate M7404 and a *cdtR* mutant in the UK isolate R20291 and confirmed their genotype by Southern hybridisation (S1 Fig). Western blot analysis showed that the *cdtR* mutants produced less CDTa and CDTb compared to the wild type and that complementation with *cdtR* in *trans* resulted in over-expression of both toxin subunits (Fig 2A and 2B). Consistent with these results, ADP-ribosyltransferase assays demonstrated that the *cdtR* mutants had significantly reduced levels of CDT activity compared to the wild type, while the complemented *cdtR* mutants showed CDT activity greater than that of the wild type strain (Fig 2D and 2E). Overall, these data show, for the first time, that CdtR is important for regulating CDT production in epidemic ribotype 027 *C*. *difficile* strains.

**Fig 3:**
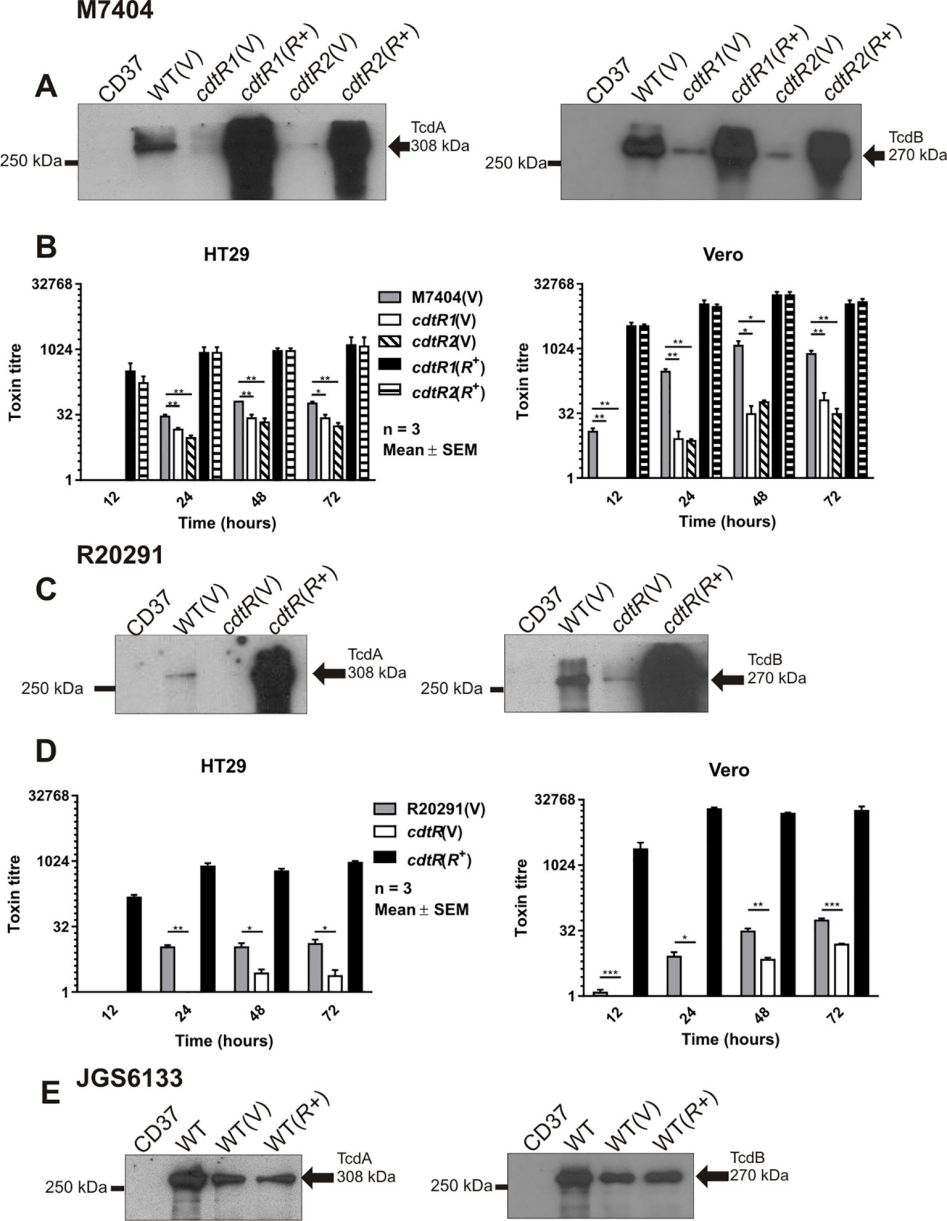
Analysis of TcdA and TcdB production. **a, c, e,** Western immunoblot using TcdA-specific and TcdB-specific antibodies with precipitated supernatant from the strains indicated. CD37 (non-toxigenic), V = vector control, *R*+ = *cdtR* complemented. Arrows indicate the 308 kDa TcdA and 270 kDa TcdB proteins. Supernatants were collected at 12, 24, 48 and 72 hours post inoculation and assayed by doubling dilution cytotoxicity assays. **b,** The panel of M7404 strains were assayed using HT29 cells and Vero cells. **d**, R20291 panel of strains assayed using HT29 cells and Vero cells. Data represent the mean ± SEM (n = 3–5). *, p ≤ 0.05; **, p ≤ 0.01; ***, p ≤ 0.001.

Unexpectedly, Western blots performed using TcdA- and TcdB-specific antibodies showed that the *cdtR* mutants produced less TcdA and TcdB than the wild type, while the complemented *cdtR* derivatives expressed high levels of both toxins (Fig 3A and 3C). Cytotoxicity assays were performed using HT29 and Vero cells to measure the activity of TcdA and TcdB, respectively, in the culture supernatants from the isogenic panel of M7404 and R20291 strains. The TcdA and TcdB activities of all of strains increased over time and, consistent with the Western blot results, showed lower activity in supernatants from the *cdtR* mutants compared to the wild type, confirming that the *cdtR* mutants were less cytotoxic *in vitro* (Fig 3B and 3D). TcdA and TcdB activity of the complemented *cdtR* mutants was consistently higher than the *cdtR* mutants and the wild type across all time points (Fig 3B and 3D). These data show that CdtR regulates the production of TcdA and TcdB in both M7404 and R20291, which is the first demonstration of a common regulator modulating the expression of all three toxins in *C*. *difficile*.

### CdtR positively regulates expression of genes from the pathogenicity locus (PaLoc) of *C*. *difficile*

**Fig 4:**
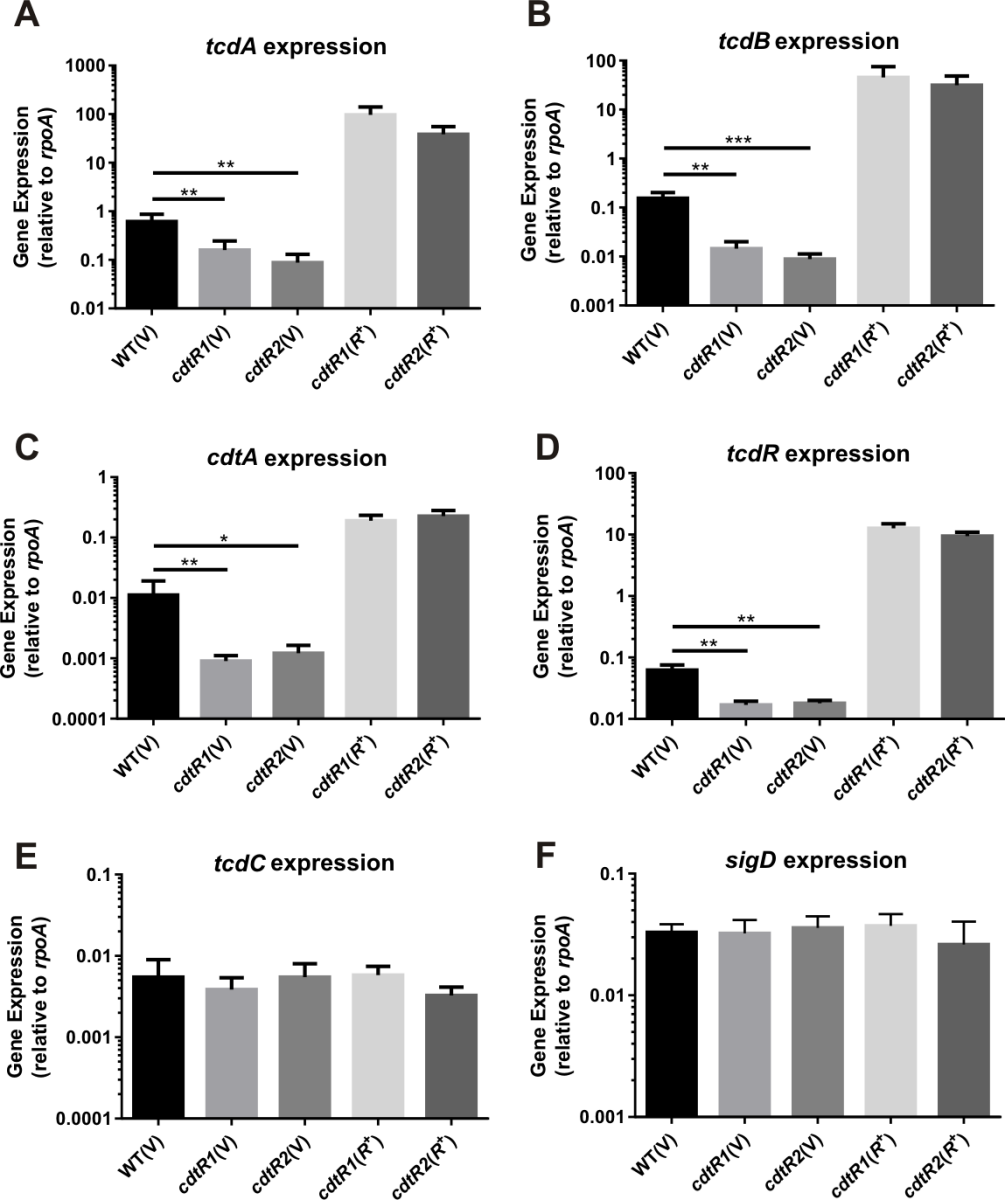
Transcriptional analysis of M7404 *cdtR* mutant and complemented strains compared to wild-type. RNA was isolated from strains for analysis of (**a**) *tcdA*, (**b**) *tcdB*, (**c**) *cdtA*, (**d**) *tcdR*, (**e**) *tcdC* and (**f**) *sigD* expression. Levels of gene expression were normalised to *rpoA*. Data represent the mean normalised gene expression ± SEM from five independent biological replicates. *, p ≤ 0.05; **, p ≤ 0.01; ***, p ≤ 0.001.

To investigate the molecular mechanism of regulation, we determined if CdtR controlled toxin production at the transcriptional level. Using the isogenic panel of M7404 strains, reverse-transcription droplet digital PCR (RT-ddPCR) analysis was employed to quantitatively compare the level of expression of each of the toxin encoding genes (*tcdA*, *tcdB*, *cdtA*) and the PaLoc-encoded toxin regulators (*tcdR*, *tcdC*). The relative transcription of all three toxin genes and *tcdR* was significantly decreased in both *cdtR* mutants compared to the wild type (Fig 4A–4D). Over-expression of *cdtR* in the complemented strains resulted in a dramatic over-expression of *tcdA*, *tcdB*, *cdtA* and *tcdR* (Fig 4A–4D). Although TcdC is predicted to be non-functional in the 027 strains [12], we analysed the expression of *tcdC* and found it to be similar in the isogenic panel of strains (Fig 4E). Previous work in strain 630Δ*erm*, a derivative of the non-027 historical strain 630 which belongs to ribotype 012, showed that the flagella synthesis regulator, SigD, is an important regulator of TcdA and TcdB production *via* the regulation of *tcdR* expression [24]. Analysis of our isogenic panel of M7404 strains showed no change in *sigD* transcription, suggesting that CdtR does not influence the expression of *sigD* in this strain background and that therefore the modulation of *tcdA* and *tcdB* expression does not occur *via* SigD (Fig 4F). Collectively, these results indicate that regulation of all three toxins by CdtR in M7404 occurs at a transcriptional level and that the regulation of *tcdA* and *tcdB* occurs *via* the upregulation of *tcdR* transcription.

### CdtR is important for modulating *C*. *difficile* virulence

**Fig 5:**
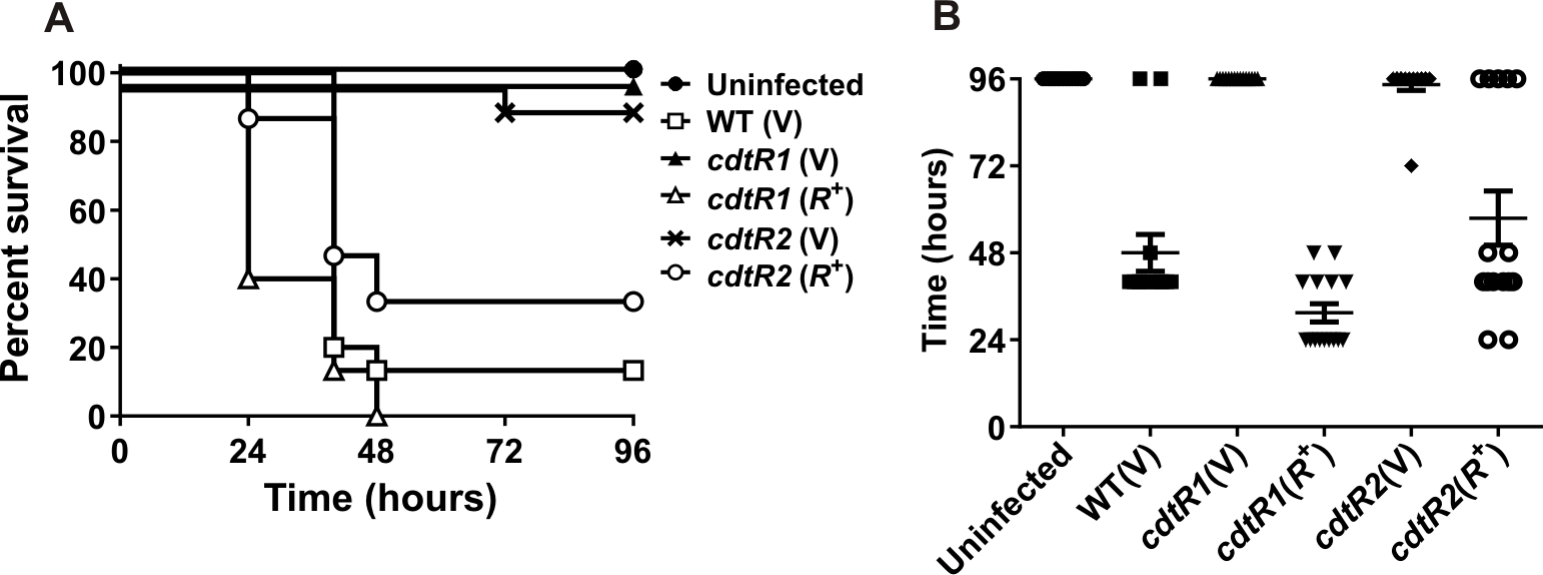
Virulence of M7404 wild-type, *cdtR* mutant and complemented strains in mice. **a**, Kaplan-Meier survival curve showing time from infection to euthanasia of mice infected with different strains of *C*. *difficile* in hours. (n = 15). **b**, Time from inoculation of mice to death in hours. Data represents mean ± S.E.M. (n = 15). Data represent the mean ± SEM ****, p ≤ 0.0001.

Our observation that CdtR increases toxin production in ribotype 027 strains has important implications for the virulence capacity of these clinically important strains. To determine if the modulatory effect of CdtR on toxin production influences *C*. *difficile* disease, we examined whether *cdtR* inactivation altered virulence in our mouse infection model [2]. It was previously shown that infection with a *cdtA* mutant of M7404 resulted in disease that was indistinguishable from the parent strain [2]. This mutant no longer produced CDT, but had an intact *cdtR* gene and continued to produce TcdA and TcdB at wild type levels. A reduction in CDT levels is therefore not likely to have a major effect on disease in our animal model. All of the mice infected with the isogenic *cdtR*-series of M7404 derivatives were colonised with *C*. *difficile* at similar levels (S2 Fig). Wild type-infected mice rapidly lost weight and the majority were euthanized 40 to 48 hours post infection in accordance with animal ethics guidelines, with a mean time to death of 48 ± 5.1 hours and a survival rate of 13% (Fig 5). Mice infected with either of the two independent *cdtR* mutants had significantly higher survival rates (Mantel-Cox log rank test, *P* < 0.0001) of 100% and 96% for *cdtR1*(V) and *cdtR2*(V), respectively, and showed no overt signs of disease nor significant weight loss (Fig 5). By comparison, mice infected with either complemented mutant, *cdtR1*(*R* ^+^) or *cdtR2*(*R* ^+^), had a wild-type virulence phenotype, with marked weight loss, and a mean time to death of 31.5 ± 2.5 hours and 57.6 ± 7.5 hours, respectively, reflected in survival rates of 0% and 33% (Fig 5).

**Fig 6:**
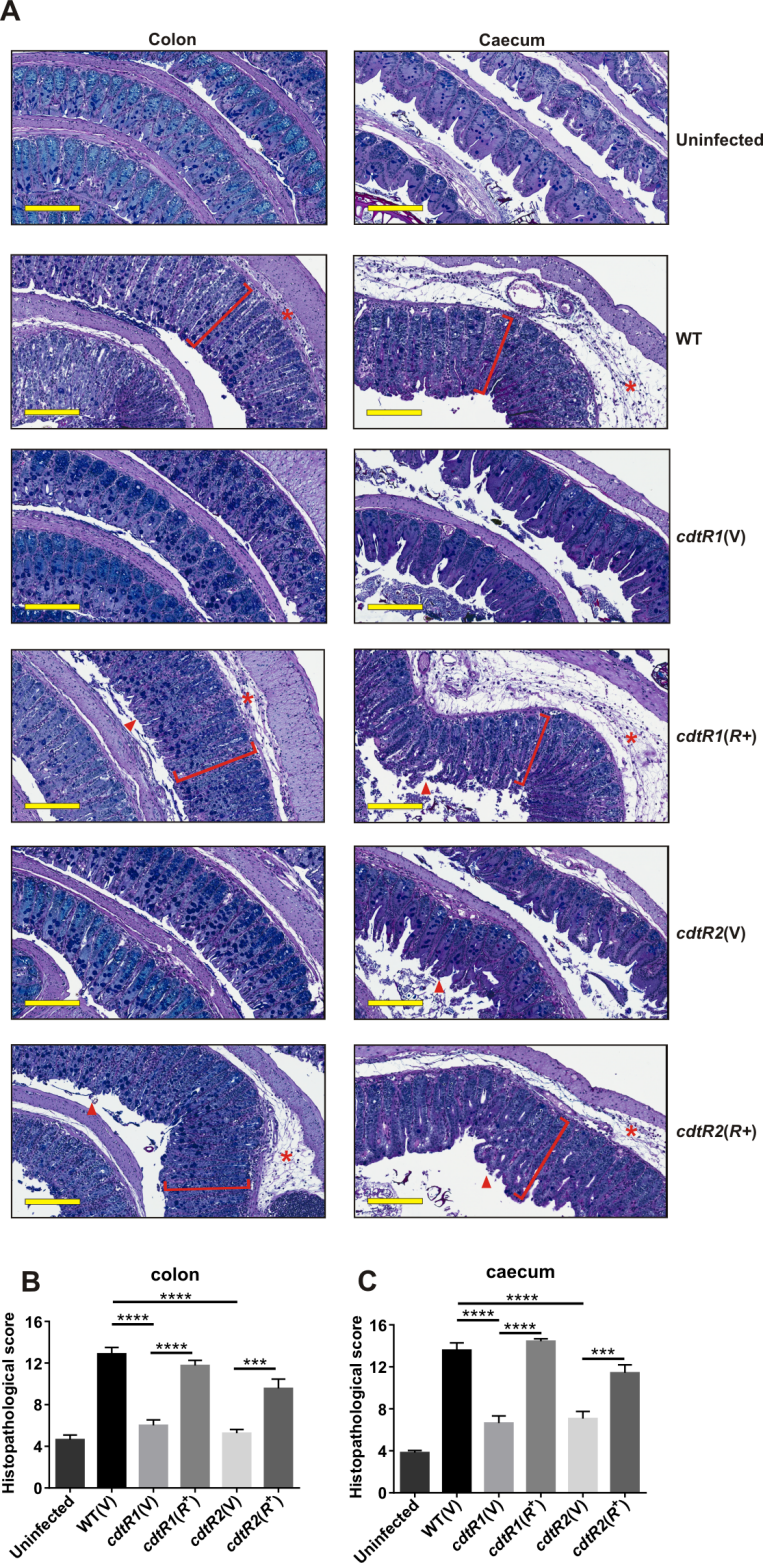
Histopathology of *C*. *difficile* infected tissues. Representative images of sections of colon and caeca collected from uninfected mice or mice infected with different strains of *C*. *difficile*, fixed and strained with PAS-Alcian blue. Red brackets ([) indicate crypt hyperplasia, arrow heads (▲) represent surface epithelial damage and asterisks (*) represent oedema and inflammation. Scale bars (200 μm) are shown in yellow. Histopathology damage scores from uninfected or infected colons (**b**) and caeca (**c**). Data represent the mean ± SEM ****, p ≤ 0.0001.

Damage to the colon and caecum of *C*. *difficile-*infected mice results from the production of TcdA and TcdB [2]. We therefore performed histopathological analysis to assess the damage to colonic and caecal tissues collected from the groups of infected and uninfected mice under study here. All tissues were de-identified and independently scored using a previously defined set of scoring parameters that included overall tissue damage, polymorphonucleocyte (PMN) influx, crypt damage and oedema [2]. Tissues of uninfected mice only had minimal surface damage to the intestinal epithelia resulting from the disruption of microbiota by antibiotic pre-treatment and tissue processing (Fig 6A) with low colon and caecum damage scores of 4.7 and 3.8, respectively (Fig 6B and 6C). By comparison, wild type-infected mice had severely inflamed tissues, with extensive damage to the epithelial surface, crypt branching and hyperplasia, goblet cell loss, significant PMN influx and mucosal and sub-mucosal oedema (Fig 6A). These histopathologies were reflected in the high damage scores of 12.9 and 13.6 for their colonic and caecal tissues, respectively (Fig 6B and 6C).

Mice infected with either *cdtR* mutant had tissue architecture similar to that seen with the uninfected mice. Very little colonic and caecal damage was observed, with some surface epithelial damage to crypts occurring and no apparent crypt hyperplasia, and little PMN influx into the mucosa (Fig 6A), with correspondingly low colonic (6.0 and 5.3) and caecal (6.6 and 7.1) damage scores (Fig 6B and 6C). However, mice infected with either *cdtR-*complemented mutant had similar levels of tissue damage to mice infected with the wild-type strain. Severe crypt damage was observed in the majority of these tissues, particularly the caeca, with crypt hyperplasia, loss of goblet cells, PMN influx and severe oedema in the mucosa and sub-mucosa (Fig 6A) and high histopathological damage scores were determined for both the colon (11.8 and 9.6) and the caecum (14.5 and 11.4) (Fig 6B and 6C).

To confirm that the reduced virulence of the *cdtR* mutants can be attributed to reduced levels of TcdA and TcdB production *in vivo*, the cytotoxicity of the intestinal contents collected from mice infected with the panel of *C*. *difficile* strains was assessed. No cytotoxicity was observed against HT29 or Vero cells by faecal samples collected from uninfected mice whereas mice infected with the wild type strain showed high levels of cytotoxicity against HT29 and Vero cells, indicating the production of TcdA and TcdB *in vivo* (S4 Fig). By comparison, samples collected from mice infected with the two *cdtR* mutants (*cdtR1*(V) and *cdtR2*(V)) showed reduced cytotoxicity against HT29 or Vero cells, indicating decreased levels of TcdA and TcdB production *in vivo* compared to the wild type strain (S4 Fig). Intestinal contents collected from mice infected with the two complemented *cdtR* mutants ((*cdtR1*(*R* ^+^) and *cdtR2*(*R* ^+^)) showed increased levels of cytotoxicity against HT29 and Vero cells, indicating restored *in vivo* TcdA and TcdB production. Collectively, the survival data, histopathological and *in vivo* cytotoxicity analysis support the hypothesis that CdtR modulates the virulence of the *C*. *difficile* ribotype 027 strain M7404 due to the role that it plays in regulating TcdA and TcdB production.

### CdtR differentially regulates toxin production in a ribotype 078 strain of *C*. *difficile*

To investigate whether TcdA and TcdB regulation by CdtR occurs in other strains, we assessed this phenotype in a ribotype 078 animal isolate, JGS6133. Ribotype 078 strains are commonly isolated from animals, but have also been implicated in severe human infections [25,26]. Our sequencing analysis revealed that the *cdtR* gene in JGS6133 contains a naturally occurring stop codon mutation at codon 322, which has been described previously in other 078 strains [27], and results in a 142 amino acid truncation that is likely to result in a non-functional protein (Fig 1B). Western blots showed that JGS6133 complemented with the full length *cdtR* gene *in trans*, and hence producing functional CdtR, had significantly more CDTa and CDTb than the wild-type and vector control strains, which produced similar amounts of both proteins (Fig 2C). Increased CDT production by the JGS6133 *cdtR* ^+^ derivative was reflected in increased toxin activity as assessed by ADP-ribosyltransferase assays (Fig 2F). By contrast, production of TcdA and TcdB was not altered in the JGS6133 *cdtR* ^+^ strain compared to the wild type (Fig 3E). This result suggests that CdtR-mediated regulation of TcdA and TcdB production is not conserved within all strains of *C*. *difficile*.

### CdtR does not regulate TcdA and TcdB production in strain 630

Derivatives of a ribotype 012 strain, designated 630, have been routinely used for the analysis of gene regulation and other phenotypes in *C*. *difficile* due to the relative ease of their genetic manipulation. Many strains of *C*. *difficile*, including strain 630, contain *cdtAB* pseudogenes within the CdtLoc (Fig 1C). These genes contain multiple frameshift mutations and stop codons and do not encode a functional CDT, but still encode a full length CdtR protein [28]. The possibility that CdtR functions to regulate other important processes, such as TcdA and TcdB production, may provide a rationale for the retention of functional CdtR in strains that carry the *cdtAB* pseudogenes. We were therefore interested in investigating CdtR-mediated toxin regulation in a strain with these pseudogenes.

To determine whether CdtR regulates TcdA and TcdB production in strains of *C*. *difficile* with *cdtAB* pseudogenes we transferred the *cdtR* complementation vector, pJIR4218, into strain 630. Western blot analysis showed that derivatives of 630 over-expressing *cdtR* had similar levels of TcdA and TcdB production as the isogenic vector control strains (S3A and S3B Fig). RT-ddPCR analysis confirmed that *cdtR* was over-expressed in these strains relative to the wild type and the vector control (S3C Fig). Taken together these results suggest that CdtR is not important for the regulation of TcdA and TcdB production in strain 630. Since CdtR is also not important for TcdA and TcdB regulation in a ribotype 078 strain, regulation of these toxins by CdtR may be specific to the ribotype 027 strains.

## Discussion

The emergence of epidemic ribotype 027 strains over a decade ago prompted several investigations of the genetic and phenotypic characteristics that may have led to the global dominance of these strains. These features may include higher sporulation rates, resistance to key antibiotics and unique aspects of toxin regulation [11,12,29–31]. The presence of a full length CdtLoc was also initially considered to be important in this regard because it encodes CDT [21,23], however, despite numerous studies, the importance of this toxin in virulence remains undefined [2,32,33]. The results of the work presented here suggest that CdtLoc, and specifically CdtR, may play an indirect but significant role in disease pathogenesis of the ribotype 027 strains by regulating TcdA and TcdB production.

Our work confirms that CdtR enhances CDT production. Strikingly, ribotype 078 strains produce CDT even though they contain a conserved mutation in *cdtR* and they have CDT activity that is not significantly different from that of strains without this mutation [27,34]. Our data supports these observations since CDT production was detected from the ribotype 078 strain JGS6133 which contains this naturally occurring *cdtR* mutation. However, CDT production from JGS6133 was enhanced when functional CdtR was expressed in this strain. Even though CdtR is not essential for CDT production, our work suggests that the presence of this regulator increases the expression of this toxin.

Although CdtR was previously shown to be an important regulator of CDT production [6] there was no evidence to suggest that it played a role in TcdA or TcdB production, particularly since the pathogenicity loci encoding these toxins are not genomically linked. Our data clearly show that CdtR is an important regulator of TcdA and TcdB production, as well as CDT, in two ribotype 027 strains, and that this regulatory capacity plays a role in virulence since inactivation of *cdtR* attenuated the virulence of strain M7404 in a mouse infection model. The results obtained with the mouse infection experiments are directly relevant to the disease-causing capacity of these strains. This is a significant finding as it is the first report of a regulatory link between the two pathogenicity loci, PaLoc and CdtLoc.

The ability to regulate toxin production through a variety of mechanisms may provide a selective advantage to *C*. *difficile* since it may allow virulence factors to be produced in response to different and specific environmental cues. Ribotype 027 strains appear to have evolved to differ in their regulatory responses in comparison to other *C*. *difficile* lineages. *C*. *difficile* regulates toxin production in response to many environmental stimuli, including metabolisable carbon sources and quorum signalling molecules, through several different regulatory proteins [20,35,36]. TcdR is the primary positive regulator of TcdA and TcdB production, while TcdC represses toxin production [12,15]. TcdR is highly conserved between strains and many regulators directly influence its expression [20,24,27,35]. Our data suggest that CdtR may regulate TcdA and TcdB production by controlling *tcdR* expression. CdtR belongs to the LytTR family of DNA binding response regulators and may function by binding to the *tcdR* promoter, thereby regulating TcdR expression and, consequently, *tcdA* and *tcdB* expression [6]. However, we could not identify canonical LytTR DNA binding sites upstream of the *tcdR*, *tcdA* or *tcdB* genes. Similarly, LytTR DNA binding sequences with the conserved sequence and spacing could not be identified within the promoters of other genes identified to regulate toxin production in *C*. *difficile* in other studies, including *sigD*, *codY* or *ccpA*. It may be that the CdtR binding sites are too dissimilar to typical LytTR sites to be identified or that CdtR does not directly bind to these regions; instead, an unidentified, CdtR-controlled intermediate regulator may be modulating PaLoc toxin gene expression.

CdtR-mediated TcdA and TcdB regulation may have specifically evolved in ribotype 027 *C*. *difficile* strains since the co-regulation of these toxins with CDT appears to be ribotype specific. CdtR does not play a similar role in other ribotypes tested here to that seen in ribotype 027 strains and this phenotype does not appear to be conserved between divergent strain backgrounds. The strains included in our assessment belong to three of the five defined evolutionary clades of *C*. *difficile*, specifically, clade 2 for ribotype 027 (M7404 and R20291), clade 5 for ribotype 078 (JGS6133) and clade 1 for ribotype 012 (630) [37]. Our observations in strain 630 are particularly relevant; many studies are performed using this isolate because it is relatively easy to genetically manipulate. It is clear from our research and other studies [10,11] that strain 630 characteristics may not always reflect those of other strains. Similar observations have been made for clade 5 ribotype 078 strains, which are genetically and phenotypically divergent from strains belonging to other clades [11,30,38,39].

Although the global regulators CodY and CcpA are conserved in strains of *C*. *difficile*, it has been shown that these regulators control toxin production experimentally only in a strain 630 background and their role in other strains, including the 027 and 078 strains, is not known [19,20]. Similarly, several flagella structural and regulatory genes, including SigD, have only been linked to toxin production in *C*. *difficile* in strain 630 [24]. While the genetic organisation of the flagella genes within the F1 and F3 flagella regions are similar in strain 630 and the 027 strains, the sequence variation in these regions is thought to contribute to their different motility phenotypes [30]. By comparison, the F3 region, which contains several genes involved in toxin regulation in strain 630, is absent in the 078 strains and is thought to explain the lack of motility in these strains [30]. It has been shown that several of the conserved flagella structural proteins encoded in the F1 and F3 flagella regions, including FliC, FliD and FlgE, are important for toxin production in strain 630 but do not contribute to toxin production in the 027 strain, R20291 [10]. Further research is required to determine if other conserved flagella genes, known to regulate toxin production in strain 630, play a similar role in 027 and 078 strain backgrounds.

To date, only one study has investigated the regulation of toxin production in an 078 strain and showed that the master sporulation regulator, Spo0A, differentially regulates toxin production in an 078 strain, two epidemic 027 strains and a strain 630 derivative [11]. Dingle et al. [40] found that strains from clade 5, including the 078 strains, carry a PaLoc similar to that found in other ribotypes but that genes outside of this region are highly divergent. It was suggested that the 078 strains may have originated from a divergent, non-toxigenic strain that obtained the PaLoc in a separate event in comparison to other toxigenic lineages. We present data supporting the concept that strains from this background have evolved different toxin regulatory mechanisms from the more commonly studied 027 strains and strain 630 derivatives.

The results presented here clearly show that modulation of *tcdA* and *tcdB* expression by CdtR may be specific to the ribotype 027 strains and is likely to be an important factor contributing to their increased virulence. Furthermore, the fluidity of the regulatory systems that control gene expression in *C*. *difficile*, exemplified by the toxin gene expression studies presented here, reflect the plasticity and dynamic nature of the *C*. *difficile* genome [37].

In conclusion, we have provided the first evidence that TcdA and TcdB production is linked to the production of CDT by a common regulatory mechanism and that CdtR acts as a global regulator of toxin production and virulence in two ribotype 027 strains. The observed differences in virulence between the ribotype 027 strains and other historical isolates have been attributed, in part, to elevated toxin production, mainly as a result of mutations in the *tcdC* gene [21–23]. Another key genetic difference identified between these strains is the possession of a full length CdtLoc [30]. Our results suggest that possession of the CdtLoc in the 027 strains enhances virulence by the CdtR-mediated up-regulation of TcdA and TcdB production. Therefore, we postulate that the ability of the epidemic ribotype 027 strains to coordinate production of all known *C*. *difficile* toxins, CDT, TcdA and TcdB, by CdtR is a key factor in the increased virulence of these strains.

## Methods

### Bacterial strains and growth conditions

All bacterial strains are defined in Table 1. Culture media were from Oxoid or Becton Dickinson (BD) and all antibiotics and supplements used are from Sigma-Aldrich, Amresco or Merck unless otherwise stated. *E*. *coli* and *B*. *subtilis* strains were cultured at 37°C in 2xYT media [41] supplemented with either chloramphenicol (25 µg/ml for *E*. *coli*; 5 μg/ml for *B*. *subtilis*) or tetracycline (10 µg/ml). *C*. *difficile* strains were cultured in HIS broth[42] or on HIS agar supplemented with 0.1% (w/v) L-cysteine and 0.375% (w/v) glucose or TY broth[1] with D-cycloserine (250 µg/ml), thiamphenicol (10 µg/ml), lincomycin (50 µg/ml) or anhydrous tetracycline (50 ng/ml), as required. *C*. *difficile* cultures were grown in a Don Whitley A35 Anaerobic Workstation in an atmosphere of 10% (v/v) H_2_, 10% (v/v) CO_2_ and 80% (v/v) N_2_ at 37°C.

**Table 1:** Bacterial strains and plasmids.

Strain or plasmid	Characteristics	Source or reference
**Strain**		
***E*. *coli***		
DH5α	F– Φ80d*lac*ZΔM15 Δ(*lacZYA-argF*) *U169 recA1 endA1 hsdR17*(r_K_^–^, m_K_^+^) *deoR thi-1 supE44 gyrA96 relA1*	Life Technologies
Top10	F–*mcrA* Δ(*mrr* ^-^ *hsdRMS* ^-^ *mcrBC*) Φ80*lac*ZΔM15 Δ*lacX74 nupG recA1 araD139* Δ(*ara-leu*)*7697 galU galK rpsL* (Str^R^) *endA1* λ^–^	Life Technologies
MM294	F^–^, *endA1*, *hsdR17*(r_K_^–^m_K_^+^), *supE44*, *thi-1*, *recA* ^*+*^	[50]
***B*. *subtilis***		
BS34A	*B*. *subtilis* donor that carries a single copy of Tn*916* on its chromosome, Tc^R^	[51]
JIR6342	BS34A carrying *cdtR* TargeTron plasmid, pJIR4153	This study
JIR6345	BS34A carrying *cdtR* complementation plasmid, pJIR4218	This study
JIR6346	BS34A carrying shuttle plasmid, pDLL24	This study
***C*. *difficile***		
M7404	Canadian BI/NAP1/027 isolate	[6]
R20291	UK BI/NAP1/027 isolate	[39]
CD37	Non-toxigenic *C*. *difficile* isolate	[52]
JGS6133	Porcine ribotype 078 isolate	[12]
DLL3094	M7404 (pDLL24)	This study
*cdtR1* (or JIR8707)	M7404 *cdtR*::TargeTron (mutant 1), Ln^R^	This study
*cdtR2* (or JIR8708)	M7404 *cdtR*::TargeTron (mutant 2), Ln^R^	This study
JIR8739	JIR8707 (pDLL24), Ln^R^, Tm^R^	This study
JIR8740	JIR8707 (pJIR4218), Ln^R^, Tm^R^	This study
JIR8741	JIR8708 (pDLL24), Ln^R^, Tm^R^	This study
JIR8742	JIR8708 (pJIR4218), Ln^R^, Tm^R^	This study
JIR8729	R20291 *cdtR*::TargeTron, Ln^R^	This study
JIR8745	JIR8729 (pDLL24), Ln^R^, Tm^R^	This study
JIR8746	JIR8729 (pJIR4218), Ln^R^, Tm^R^	This study
JIR8747	R20291 (pDLL24), Tm^R^	This study
JIR8733	JGS6133 (pDLL24), Tm^R^	This study
JIR8735	JGS6133 (pJIR4218)	This study
**Plasmid**		
pDLL4	*C*. *difficile* shuttle vector allows plasmid to be mobilised by Tn916, Tm^R^	[12]
pDLL24	pDLL4 carrying *lacZα*, Cm^R^	This study
pJIR4218	pDLL24 carrying *cdtR* and its promoter, Cm^R^	This study
pDLL45	pMTL9361 derivative with *Hin*dIII and *Bsr*GI sites removed from *rep*; Tm^R^	This study
pDLL55	Derivative of pDLL45 carrying *lacZα*; Tm^R^	This study
pJIR4135	Group II intron of pDLL45 retargeted to insert between codons 288/289 of the *cdtR* gene, Cm^R^	This study
pJIR4153	pDLL55 (*Stu*I/*Hin*dIII)Ω pJIR4135 group II intron (*Stu*I/*Hin*dIII)	This study

### Polymerase Chain Reaction (PCR)

All oligonucleotide primers are listed in Table 2. PCR cycling conditions (unless otherwise stated) were as follows: initial denaturation step at 94°C for 4 min, followed by 30 cycles of denaturation at 94°C for 30 sec, an annealing step at 50°C for 30 sec and an extension step at 72°C for 1 min per 1 kb. A final extension step was performed at 72°C for 10 minutes. PCRs were performed with Phusion DNA polymerase (New England Biolabs) and 2x Failsafe PCR buffer E (Epicentre Biotechnology). Splice-overlap extension (SOE)-PCR to re-target the Targetron was performed as described in the TargeTron Gene Knockout System users guide (Sigma-Aldrich) with modifications as previously described [43].

**Table 2:** Oligonucleotide primers.

**Primer**	**Sequence (5’- 3’)**	**Use**
JRP5448	AAAAAAGCTTATAATTATCCTTATAAAACCATTTCGTGCGCCCAGATAGGGTG	*cdtR*-288a-IBS (+)
JRP5449	CAGATTGTACAAATGTGGTGATAACAGATAAGTCCATTTCTATAACTTACCTTTCTTTGT	*cdtR*-288a-EBS1d (-)
JRP5450	TGAACGCAAGTTTCTAATTTCGGTTTTTTATCGATAGAGGAAAGTGTCT	*cdtR*-288a-EBS2 (+)
JRP3867	CGAAATTAGAAACTTGCGTTCAGTAAAC	EBS universal (-)
JRP5632	AAAAGGATCCCTTCTATAATTAGAAGTTAAATAATTCTTC	Amplify *cdtR* gene and upstream region, introduces *Bam*HI site (+)
JRP5633	AAAACTGCAGGAGACATCTCTTTTTTCTATTTATTATG	Amplify *cdtR* gene and upstream introduces *Pst*I site (-)
DLP458	TAATAAAAATACTGCCCTCGACAAA	*tcdA*-specific for RT-ddPCR (+)
DLP459	ATAAATTGCATGTTGCTTCATAACT	*tcdA*-specific for RT-ddPCR (-)
JRP6107	GCTATTAGCGAGGATAACGATTTC	*tcdB*-specific for RT-ddPCR (+)
JRP6108	CTTTCCTAGTTCCATCATAAATCTACCA	*tcdB*-specific for RT-ddPCR (-)
JRP2443	CAAGAAATAACTCAGTAGATGATTTGCAA	*tcdR*-specific for RT-ddPCR (+)
JRP2444	TCTCCCTCTTCATAATGTAAAACTCTACTAAG	*tcdR*-specific for RT-ddPCR (-)
JRP6104	AGCACAAAGGATATTGCTCTACT	*tcdC*-specific for RT-ddPCR (+)
JRP6105	AAATGACCTCCTCATGGTCTTC	*tcdC*-specific for RT-ddPCR (-)
JRP3845	TGCAATACTACTTACAAGGCTCCTATAGA	*cdtA*-specific for RT-ddPCR (+)
JRP3846	TCTTTCCCATTCTTTAGCCTTTTC	*cdtA*-specific for RT-ddPCR (-)
JRP6238	GATGCATGCTTTATTCGTGTACATA	*cdtR*-specific for RT-ddPCR (+)
JRP6239	CGACATATATGGCCATTACTCATT	*cdtR*-specific for RT-ddPCR (-)
JRP2285	GGATGATATGATGAAGGTTAGAAACCT	*rpoA*-specific for RT-ddPCR (+)
JRP2286	CCCAATCCAAGTTCTTCTAGTTTTTG	*rpoA*-specific for RT-ddPCR (-)

(+) = forward primer, (-) = reverse primer

### Isolation and manipulation of nucleic acids

Plasmid DNA was isolated from *E*. *coli*, *B*. *subtilis* and *C*. *difficile* using QIAprep spin miniprep columns (Qiagen) following the manufacturer’s instructions. Genomic DNA was isolated from *C*. *difficile* as previously described [44]. Standard methods of DNA digestion, modification and ligation were used. DNA sequencing was carried out using BigDye Terminator v3.1 Ready Reaction Mix (Applied Biosystems) following the manufacturer’s instructions. Sequencing reactions were resolved on an Applied Biosystems 3730 DNA Analyzer. Sequences were analysed using ContigExpress (Invitrogen).

### Construction of recombinant plasmids

All plasmids are outlined in Table 1. Construction of the *cdtR* TargeTron plasmid was performed as previously described, with some modifications [43]. Briefly, the group II intron from pDLL45 was retargeted by SOE-PCR to insert between nucleotides 288 and 289 of the *cdtR* gene using the primer pairs JRP5448 and JRP3867 and JRP5449 and JRP5450 (Table 2) to generate a 350 bp product, which was digested with *Bsr*GI and *Hin*dIII and cloned into the corresponding sites of pDLL45, resulting in pJIR4135. A *Stu*I-*Hin*dIII fragment was then sub-cloned from pJIR4135 into the corresponding sites of pDLL55, resulting in pJIR4153.

The *cdtR* complementation plasmid was constructed by PCR amplifying the *cdtR* gene and approximately 300 bp of its promoter region from *C*. *difficile* M7404 using the primers JRP5632 and JIR5633 (Table 2). The resulting 1.1 kb fragment was purified using a PCR purification kit (Qiagen) following the manufacturer’s instructions, digested with *Bam*HI and *Pst*I and cloned into the corresponding sites of pDLL24, resulting in pJIR4218.

### Transfer of plasmid DNA into *C*. *difficile* by conjugation

Plasmid DNA was introduced into the *B*. *subtilis* conjugative donor strain BS34A as previously described [45]. The resulting strain was used as the donor for the conjugative transfer of plasmid DNA into *C*. *difficile* strains as before [11].

### Isolation of *cdtR* mutants

*C*. *difficile cdtR* mutants were isolated using the method previously described [11] and confirmed by PCR and Southern hybridisation analysis. Complementation of the mutation was achieved using the *cdtR* complementation plasmid, pJIR4218. The cloning vector, pDLL24, was transferred into the *cdtR* mutant and the wild-type strain to construct vector (v) controls.

### Detection of TcdA, TcdB, CDTa and CDTb by western blotting

Toxins were partially purified and concentrated eight-fold from 72 hour *C*. *difficile* TY culture supernatants by methanol-chloroform precipitation [11]. Protein concentrations were determined using the BCA protein assay kit (Pierce) as per the manufacturer’s instructions. Concentrated supernatant proteins (10 µg) were separated by 10% (v/v) sodium dodecyl sulfate-polyacrylamide gel electrophoresis (SDS-PAGE) [46] and transferred onto a nitrocellulose membrane (Whatman). Membranes were analysed as previously described [43]. TcdA and TcdB were detected using TcdA-specific monoclonal and TcdB-specific polyclonal antibodies (tgcBIOMICS), respectively. CDTa and CDTb were detected, respectively, using a CDTa-specific antibody and *C*. *perfringens* Ib-specific antibody that is cross reactive with CDTb [47]. CDTa, CDTb and TcdB-bound antibodies were detected using horseradish peroxidase conjugated anti-rabbit goat antibodies (Millipore) and TcdA-bound antibodies were detected using anti-mouse goat antibodies (Millipore). The Western Lightning Chemiluminescence reagent kit (Perkin-Elmer) was used to detect the bands, which were visualised by exposure to X-ray film or on a BioRad ChemiDoc XRS^+^ system.

### ADP-ribosyltransferase assays

Toxins were partially purified from culture supernatants by precipitation with 70% ammonium sulphate as described previously [6]. ADP ribosyltransferase assays were performed as previously described [48]. Briefly, precipitated supernatant protein (50 µg) was incubated for 60 minutes at 37°C with 10 µg of actin in assay buffer (20 mM Tris-HCl pH 7.5, 1 µM dithiothreitol (DTT), 40 µM ATP, 40 µM CaCl_2_, 5 µM MgCl_2_) and 10 µM of biotinylated NAD^+^ (Trevigen). The reaction was heat inactivated at 95°C for 5 minutes in 4x SDS sample buffer (240 mM Tris-Cl (pH 6.8), 40% glycerol (v/v), 8% SDS (w/v), 5% (v/v) 2-mercaptoethanol, 0.05% (v/v) bromophenol blue and separated by 10% SDS-PAGE. Proteins were transferred onto a nitrocellulose membrane and biotinylated proteins were detected with horseradish peroxidase-conjugated streptavidin (GE Healthcare Life Sciences) and the Western Lightning Chemiluminescence reagent kit (Perkin-Elmer), following the manufacturer’s instructions. Relative band intensities were determined by densitometry using Image Lab Software (Bio-Rad). Data were analysed using GraphPad Prism 6 and statistical significance assessed using an unpaired t-test with a 95% confidence interval.

### Vero and HT29 cell cytotoxicity assays

*C*. *difficile* strains were grown overnight in 20 ml of HIS broth with thiamphenicol and lincomycin, as required. The cultures then were used to inoculate 50 ml of TY broth with selection, such that each culture had a starting OD_600_ of approximately 0.05. Aliquots (5 ml) were taken at 12, 24, 48 and 72 hours, pelleted by centrifugation (10,000 *g*, 10 min, room temperature) and the supernatants filter sterilised through 0.45 µM and 0.2 µM filters (Sartorius). Supernatants were stored on ice until use. Vero cell and HT29 cell cytotoxicity assays were performed using the filtered *C*. *difficile* supernatants as previously described [43]. The levels of TcdA and TcdB produced by the *C*. *difficile* strains *in vivo* was assessed by determining the cytotoxicity of the intestinal contents collected 24 hours post infection against HT29 and Vero cells. Intestinal samples were resuspended in 100 mg/ml in PBS, diluted one in eight, filter sterilised and applied to Vero and HT29 cells, as described previously [2]. The endpoint (toxin titre) was scored as the last dilution with 100% cytopathic effect (CPE). Data were analysed using GraphPad Prism 6 and statistical significance assessed using an unpaired t-test with a 95% confidence interval.

### RT-ddPCR analysis of *C*. *difficile* gene expression

Total RNA was extracted using TRIzol® (Life Technologies) following the manufacturer’s instructions. Forty ml of *C*. *difficile* TY broth cultures with an OD_600_ of approximately 0.3 for *tcdC* and *cdtA* expression analysis, and 10 ml of TY broth culture grown to OD_600_ of approximately 1.8 for *tcdA*, *tcdB* and *tcdR* expression analysis, were used. A total of 200 ng of RNA was converted to cDNA using SuperScript III Reverse Transcriptase (Life Technologies), following the manufacturer’s instructions. Transcript levels were quantified using the QX200 Droplet Digital PCR System (BioRad) using QX200 ddPCR EvaGreen Supermix and 0.1–5 µg of total cDNA and specific primers (Table 2) at a concentration of 200 nM. Transcription levels of each gene was normalised to transcription levels of the housekeeping gene *rpoA*. Data were analysed using GraphPad Prism 6 and statistical significance assessed using a Mann Whitney U test.

### *C*. *difficile* virulence trials

Groups of five male six to eight week old C57BL/6 mice were used in *C*. *difficile* virulence trials as previously described [2], except mice were switched back to plain drinking water on the day of infection. Mice were administered 10^6^ *C*. *difficile* spores by oral gavage and were humanely euthanised at the onset of severe disease or at the end of the experiment, as previously defined [2]. Animal handling and experimentation were performed in accordance with institutional guidelines (Monash University animal ethics committee numbers MARP/2014/135 and SOBSB/M/2010/25). Faecal samples were taken daily to monitor *C*. *difficile* shedding using HIS agar supplemented with 0.1% (w/v) cysteine, 0.1% (w/v) taurocholate, 0.375% (w/v) glucose, 250 µg/ml D-cycloserine, 8 µg/ml cefoxitin, 10 µg/ml erythromycin, 12 µg/ml norfloxacin, 32 µg/ml moxalactam. Data were analysed using GraphPad Prism 6 and statistical significance assessed using a log-rank (Mantel-Cox) test.

### Histology

The entire colon and caecum were collected from each mouse and Swiss-rolled [49] prior to fixation to allow for cross-sectional examination of the entire length of the colon. Tissues were stained with PAS-Alcian blue and histopathological assessment of damage and scoring of tissues was performed blind by independent observers using a previously defined set of parameters [2].

## Supporting Information

S1 Fig Confirmation of M7404 and R20291 *cdtR* mutants by Southern hybridisation analysis. Schematic diagram of the *cdtR* genomic location and surrounding genes in (**a**) wild-type M7404 or R20291 and (**b**) TargeTron-derived *cdtR* insertion mutants. Southern hybridisation using a *cdtR* specific probe (red) showed a size increase from a 3.2 kb *Ava*II fragment in the wild type (black arrow) to a 5.0 kb *Ava*II fragment in the independent *cdtR* mutants (white arrow) in both (**c**) M7404 and (**e**) R20291 strain backgrounds. Hybridization of an *ermB* probe (purple) to a 5.0 kb *Ava*II fragment (black arrow) in the (**d**) M7404 *cdtR* mutants and (**f**) R20291 *cdtR* mutant confirmed the TargeTron insertion.
(TIF)

S2 Fig Colonisation of mice infected with *C*. *difficile* wild type, *cdtR* mutant and complemented strains. Colonisation efficiencies are shown as total colony forming unit (CFU) of *C*. *difficile* isolated per gram of faeces collected from mice at 24 and 48 hours. Mice surviving beyond 48 hours had similar levels of colonisation. Data represent the mean ± SEM (n = 6–15).
(TIF)

S3 Fig Analysis of TcdA and TcdB production in 630 stain derivatives over-expressing CdtR. Western immunoblots were performed using precipitated supernatant proteins from the CD37 non-toxigenic strain, two 630 vector control strains and two 630 strains carrying the *cdtR* ^+^ complementation vector, pJIR4218, and detected using antibodies specific for (**a**) TcdA and (**b**) TcdB. **c**, Expression of *cdtR* in 630, 630 carrying the vector control and 630 carrying the *cdtR* ^+^ complementation vector normalised to *rpoA* expression.
(TIF)

S4 Fig *In vivo* cytotoxicity of *C*. *difficile* wild type, *cdtR* mutant and complemented strains. Faecal samples collected from uninfected and *C*. *difficile* infected mice 24 hours post infection were assayed for cytotoxicity by doubling dilution cytotoxicity assays using (**a**) HT29 cells and (**b**) Vero cells. Data represent the mean ± SEM (n = 5).
(TIF)
