# Supplementary figures and images for: CdtR Regulates TcdA and TcdB Production in Clostridium difficile
Source: PLoS Pathog. 2016 Jul 14;12(7):e1005758. doi: 10.1371/journal.ppat.1005758 (PMC4944984; doi:10.1371/journal.ppat.1005758)

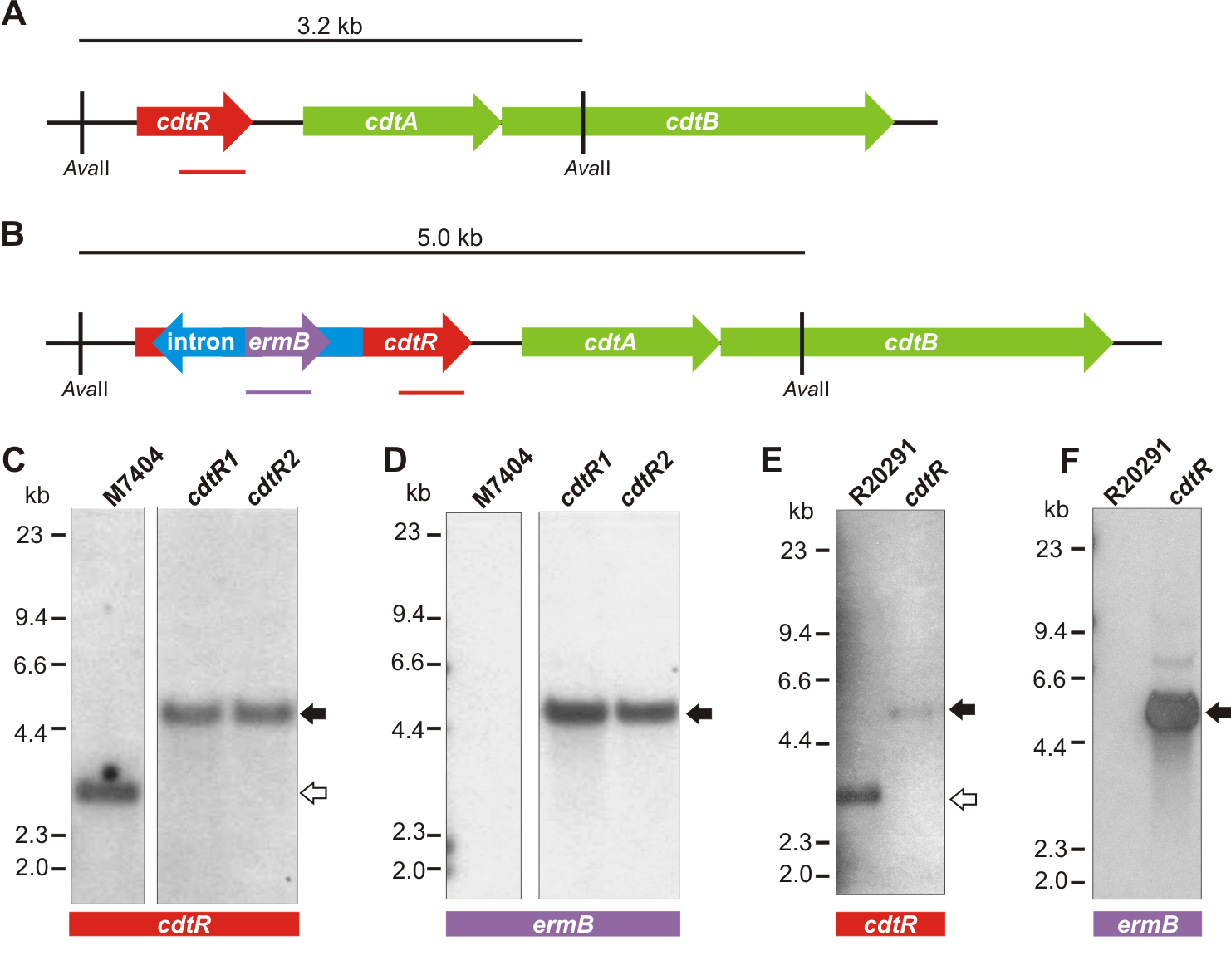

Supplement: S1 Fig — Schematic diagram of the cdtR genomic location and surrounding genes in (a) wild-type M7404 or R20291 and (b) TargeTron-derived cdtR insertion mutants. Southern hybridisation using a cdtR specific probe (red) showed a size increase from a 3.2 kb AvaII fragment in the wild type (black arrow) to a 5.0 kb AvaII fragment in the independent cdtR mutants (white arrow) in both (c) M7404 and (e) R20291 strain backgrounds. Hybridization of an ermB probe (purple) to a 5.0 kb AvaII fragment (black arrow) in the (d) M7404 cdtR mutants and (f) R20291 cdtR mutant confirmed the TargeTron insertion. (TIF) [file ppat.1005758.s001.tif]

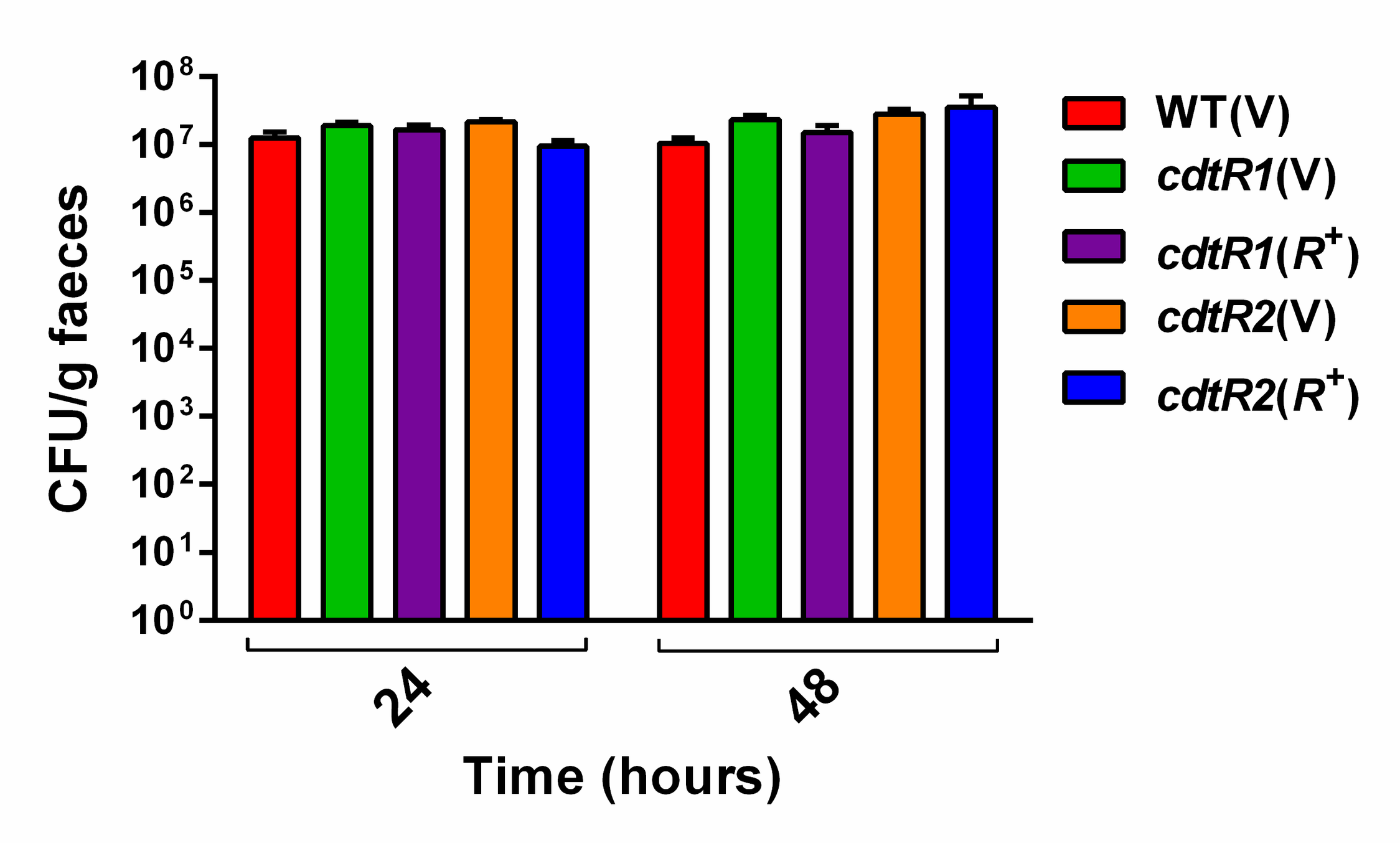

Supplement: S2 Fig — Colonisation efficiencies are shown as total colony forming unit (CFU) of C. difficile isolated per gram of faeces collected from mice at 24 and 48 hours. Mice surviving beyond 48 hours had similar levels of colonisation. Data represent the mean ± SEM (n = 6–15). (TIF) [file ppat.1005758.s002.tif]

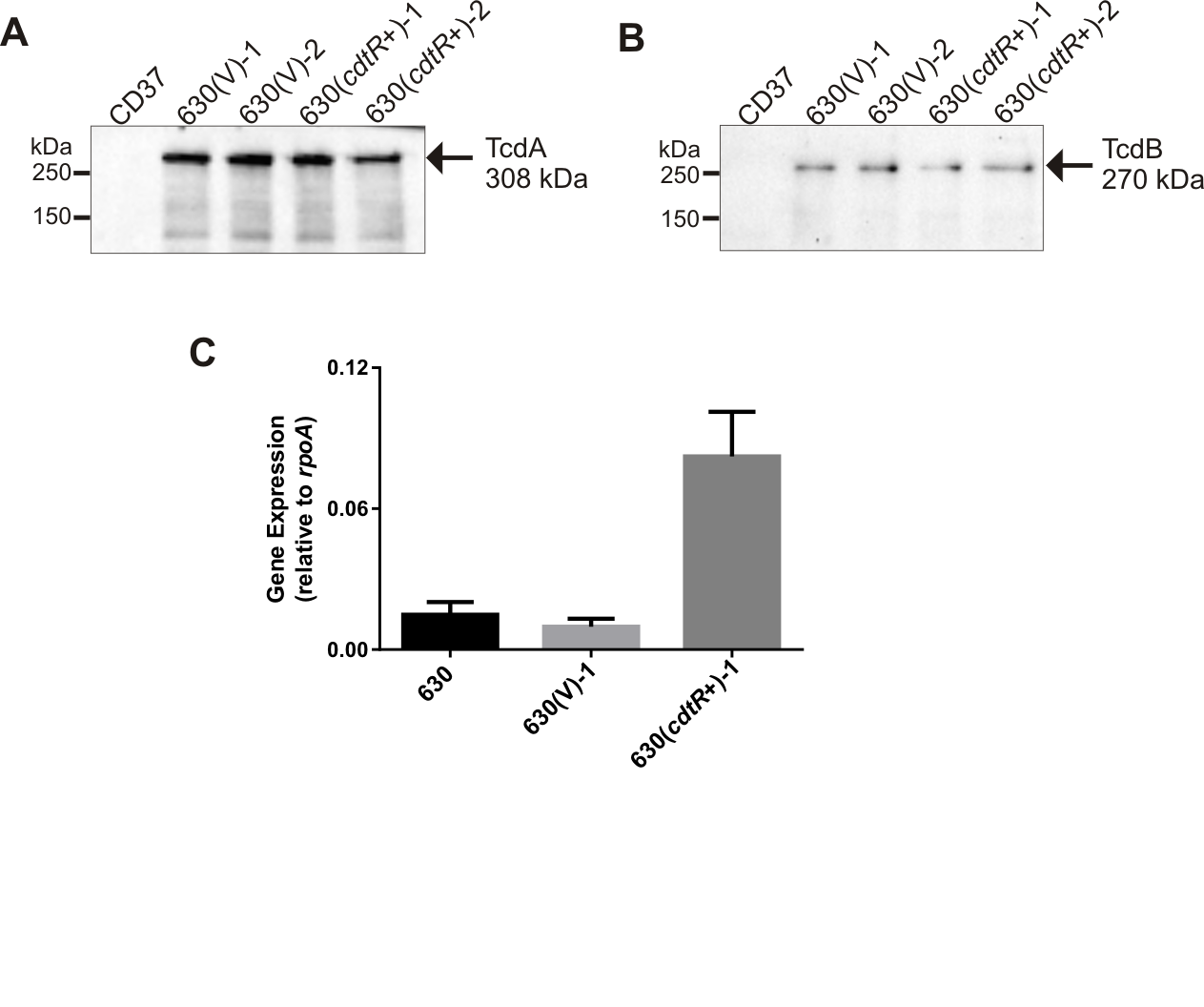

Supplement: S3 Fig — Western immunoblots were performed using precipitated supernatant proteins from the CD37 non-toxigenic strain, two 630 vector control strains and two 630 strains carrying the cdtR + complementation vector, pJIR4218, and detected using antibodies specific for (a) TcdA and (b) TcdB. c, Expression of cdtR in 630, 630 carrying the vector control and 630 carrying the cdtR + complementation vector normalised to rpoA expression. (TIF) [file ppat.1005758.s003.tif]

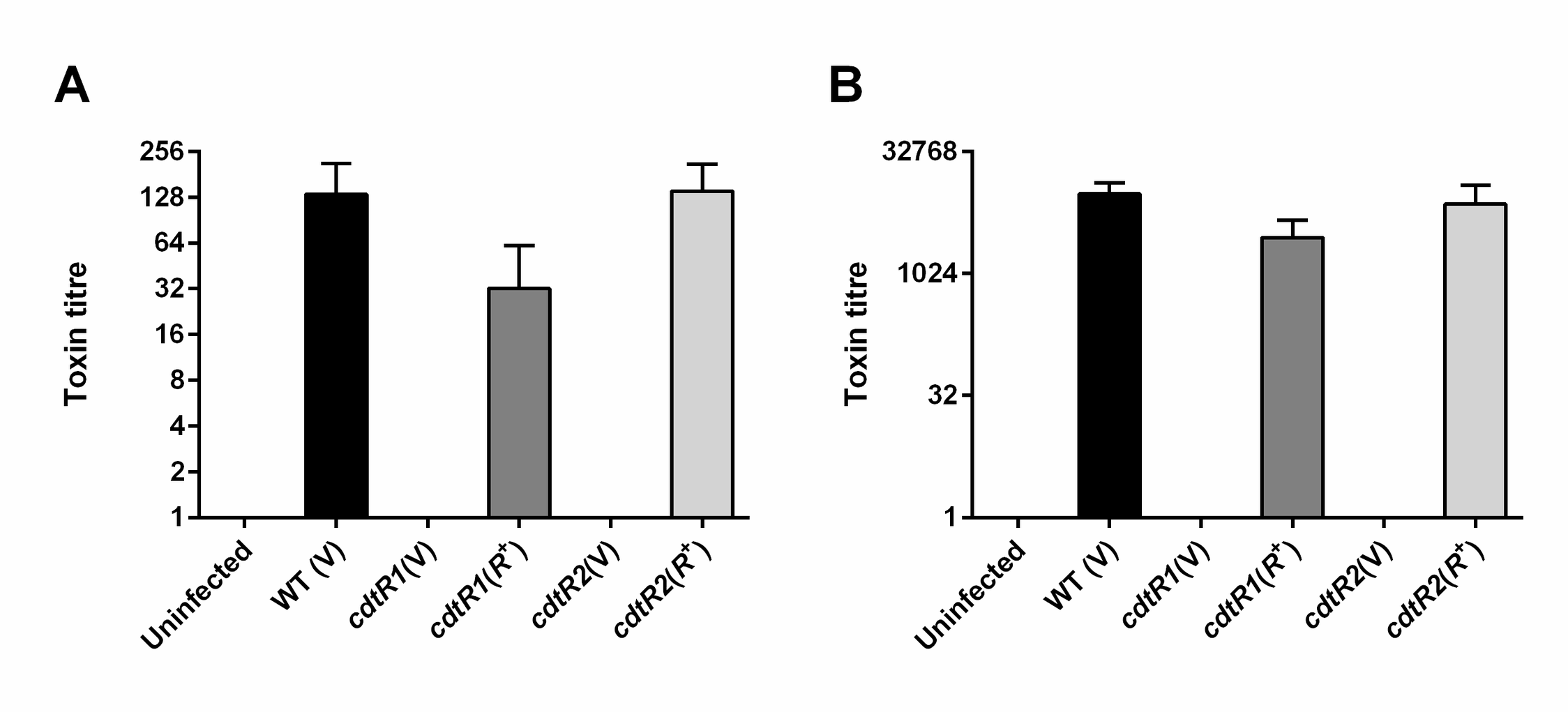

Supplement: S4 Fig — Faecal samples collected from uninfected and C. difficile infected mice 24 hours post infection were assayed for cytotoxicity by doubling dilution cytotoxicity assays using (a) HT29 cells and (b) Vero cells. Data represent the mean ± SEM (n = 5). (TIF) [file ppat.1005758.s004.tif]
